# Recent advances in ZBP1-derived PANoptosis against viral infections

**DOI:** 10.3389/fimmu.2023.1148727

**Published:** 2023-05-16

**Authors:** SuHyeon Oh, SangJoon Lee

**Affiliations:** Department of Biological Science, Ulsan National Institute of Science and Technology (UNIST), Ulsan, Republic of Korea

**Keywords:** interferon, ZBP1, inflammasome, virus, pyroptosis, apoptosis, necroptosis, PANoptosis

## Abstract

Innate immunity is an important first line of defense against pathogens, including viruses. These pathogen- and damage-associated molecular patterns (PAMPs and DAMPs, respectively), resulting in the induction of inflammatory cell death, are detected by specific innate immune sensors. Recently, Z-DNA binding protein 1 (ZBP1), also called the DNA-dependent activator of IFN regulatory factor (DAI) or DLM1, is reported to regulate inflammatory cell death as a central mediator during viral infection. ZBP1 is an interferon (IFN)-inducible gene that contains two Z-form nucleic acid-binding domains (Zα1 and Zα2) in the N-terminus and two receptor-interacting protein homotypic interaction motifs (RHIM1 and RHIM2) in the middle, which interact with other proteins with the RHIM domain. By sensing the entry of viral RNA, ZBP1 induces PANoptosis, which protects host cells against viral infections, such as influenza A virus (IAV) and herpes simplex virus (HSV1). However, some viruses, particularly coronaviruses (CoVs), induce PANoptosis to hyperactivate the immune system, leading to cytokine storm, organ failure, tissue damage, and even death. In this review, we discuss the molecular mechanism of ZBP1-derived PANoptosis and pro-inflammatory cytokines that influence the double-edged sword of results in the host cell. Understanding the ZBP1-derived PANoptosis mechanism may be critical for improving therapeutic strategies.

## Introduction

### Innate immunity and virus

Innate immunity is the first line of defense against viral infections in host cells. The pro-inflammatory response of innate immunity induces the migration of immune cells, including macrophages and neutrophils, to remove infectious agents (1, 2). The innate immune system is activated by the viral pathogen-associated molecular patterns (PAMPs) and damage-associated molecular patterns (DAMPs) by pattern recognition receptors (PRRs), such as Toll-like receptors (TLRs), retinoic acid-inducible gene I (RIG-I)-like receptors (RLRs), nucleotide-binding oligomerization domain (NOD)-like receptor family proteins (NLRs), absent in melanoma 2 (AIM2), and Z-DNA binding protein 1 (ZBP1). For example, several TLRs are involved in the detection of β-coronaviruses. TLR7 senses severe acute respiratory syndrome coronavirus (SARS-CoV), Middle East respiratory syndrome coronavirus (MERS-CoV) (3), and murine hepatitis virus (MHV) (4), and TLR2 senses SARS-CoV-2 (5). RIG-I senses viral RNA, including IAV (6–9) and hepatitis C virus (HCV) (10). Subsequently, TLR- and RLR-mediated signaling leads to the secretion of type 1 interferons (IFNs), that stimulate the expression of IFN-stimulated genes (ISGs) in infected and neighboring cells, thereby inducing an antiviral state. In particular, some PRRs, including NLRs and AIM2, assemble a large protein complex known as inflammasome, comprising a sensor, an adaptor, and an effector. They are assembled after sensing viral infections and activate the programmed cell death (PCD) pathway. The most well-established PCDs are pyroptosis, apoptosis, and necroptosis. These PCD pathways are activated against various viral infections to remove infected cells and suppress viral spread. Some viruses derive the crosstalk between the multiple PCD pathways known as PANoptosis, including IAV (11–14) and HSV1 (15–18) infection. PANoptosis occurs *via* PANoptosome, wherein the key molecules of pyroptosis, apoptosis, and necroptosis simultaneously interact with each other (14, 18–22). In this review, we summarize the molecular mechanisms of each PCD and PANoptosis against viral infection.

### ZBP1

At first, ZBP1 was considered as the cytosolic DNA sensor (23, 24). However, *Zbp1^–/–^* and wild type (WT) mice displyed a similar phenotype in B-DNA-induced innate immune activation (23–25). ZBP1 comprises three parts: the N-terminal Z-DNA binding domain (ZBD), the receptor-interacting protein homotypic interaction motifs (RHIM), and the C-terminal signal domain (SD) (26–29). The N-terminal ZBD, also called the Zα1 and Zα2, binds to the left-handed helical Z-conformation nucleic acid (Z-NA) (30, 31). Zα domains, particularly the Zα2 domain, are known to play a critical role in the activation of PCDs (18, 30, 32–34). For example, the deficiency of the Zα domains or Zα2 alone limits ZBP1-RIPK3-mediated inflammatory cell death after IAV infection (30, 34). In addition to Zα domains, ZBP1 has two RHIMs that interact with other RHIMs in RIPK1 and RIPK3 (26, 30, 35, 36). The C-terminal SD of ZBP1 participates in the type 1 IFN response induced by ZBP1 (29).

ZBP1 has recently been shown to act as a central regulator of PANoptosis, by defending against viral infections, such as IAV (11, 34) and HSV1 (18). In contrast, some viruses cause cell death that severely impacts host health. For example, SARS-CoV-2, the causative virus of coronavirus disease 19 (COVID-19), activates multiple inflammatory cell death pathways and induces the hyperactivation of cytokine secretion, which results in severe symptoms (32, 37–39). Thus, the regulation of the adverse mechanisms of PCD is essential for protecting the host from death.

### ZBP1-NLRP3 inflammasome

The inflammasome mediates pyroptosis by forming a large protein complex after sensing PAMPs and DAMPs. Inflammasomes induce the activation of protease enzyme families such as caspase-1, 4, 5, and 11, which process GSDMD and release the N-terminus to oligomerize and form pores in the plasma membrane after their cleavage (40–42). Activated caspase-1 cleaves the end to release IL-1β and IL-18. There are five well-known inflammasome sensors: NLRP1 (43), NLRP3 (44–46), NLRC4 (47, 48), AIM2 (49–52), and pyrin (53). Some other innate sensors have also been reported to initiate inflammasome assembly under specific conditions, such as NLRP6 (54), NLRP9 (55), NLRP12 (56), interferon-γ-inducible protein 16 (IFI16) (57), RIG-I (58), and myxovirus resistance protein A (MxA) (1). These sensors interact with apoptosis-associated speck-like protein containing CARD (ASC), an adaptor molecule, to activate caspase-1. Among them, NLRP3 has been extensively studied in various stimuli, from endogenous danger signals (44–46) to gram-positive (59) and gram-negative bacteria (60–62). In addition, NLRP3 senses RNA viruses, IAV (11, 63–66), and West Nile virus (WNV) (67), in addition to DNA viruses, HSV-1 (68, 69) to activate the antiviral immune response.

Two signals are required to activate the NLRP3 inflammasome. First, the priming signal from stimuli promotes the NF-κB and ERK pathways, which may elevate the gene expression of inflammasome components and manage post-translational modifications of NLRP3, such as ubiquitination (70), phosphorylation (71, 72), and SUMOylation (73). Subsequently, the activation signal stimulates NLRP3 activation, which may be due to specific cellular stress patterns such as K^+^ efflux (74, 75) and mitochondrial dysfunction (76, 77). The activated NLRP3 inflammasome contains NLRP3, caspase-1, and ASC and facilitates IL-1β maturation (78). Notably, regardless of the presence of stimuli, ASC specks can be released into the extracellular space and oligomerized in the neighboring macrophages *via* a prion-like mechanism in *Arf6^–/–^* macrophages (79).

Recently, the interaction between ZBP1 and NLRP3 inflammasome has been revealed in several viral infections. ZBP1 detects IAV infection and activates the NLRP3 inflammasome to induce inflammatory cell death (11, 30, 80). And NLRP3 inflammasome activation was diminished during IAV infection in *Zbp1^–/–^* bone marrow-derived macrophages (BMDMs) (11). In addition, the Zα2 domain of ZBP1 influences the activation of NLRP3 inflammasome and PANoptosis (30). ZBP1 can interact with IAV nucleoprotein (NP), polymerase subunit PB1, and IAV Z-RNA (11, 34), thereby activating the NLRP3 inflammasome *via* the RIPK1-RIPK3-caspase-8 axis (11). Overall, these results indicate that ZBP1 is an essential regulator of the NLRP3 inflammasome in response to viral infection.

In this review, we summarize the role of ZBP1 as an essential regulator of innate immune response and cell death during viral infection. Herein, we describe how ZBP1 senses the entry of viral Z-RNA and stimulates the inflammatory cell death pathway. We also describe a newly emerging concept of inflammatory cell death, PANoptosis, which leads to host survival when balanced or fetal symptoms and even death when exacerbated, which may be a decisive target in various viral diseases.

## ZBP1 and PCDs

### ZBP1 senses viral genome and plays a key role as a central mediator of PCDs

**Figure 1:**
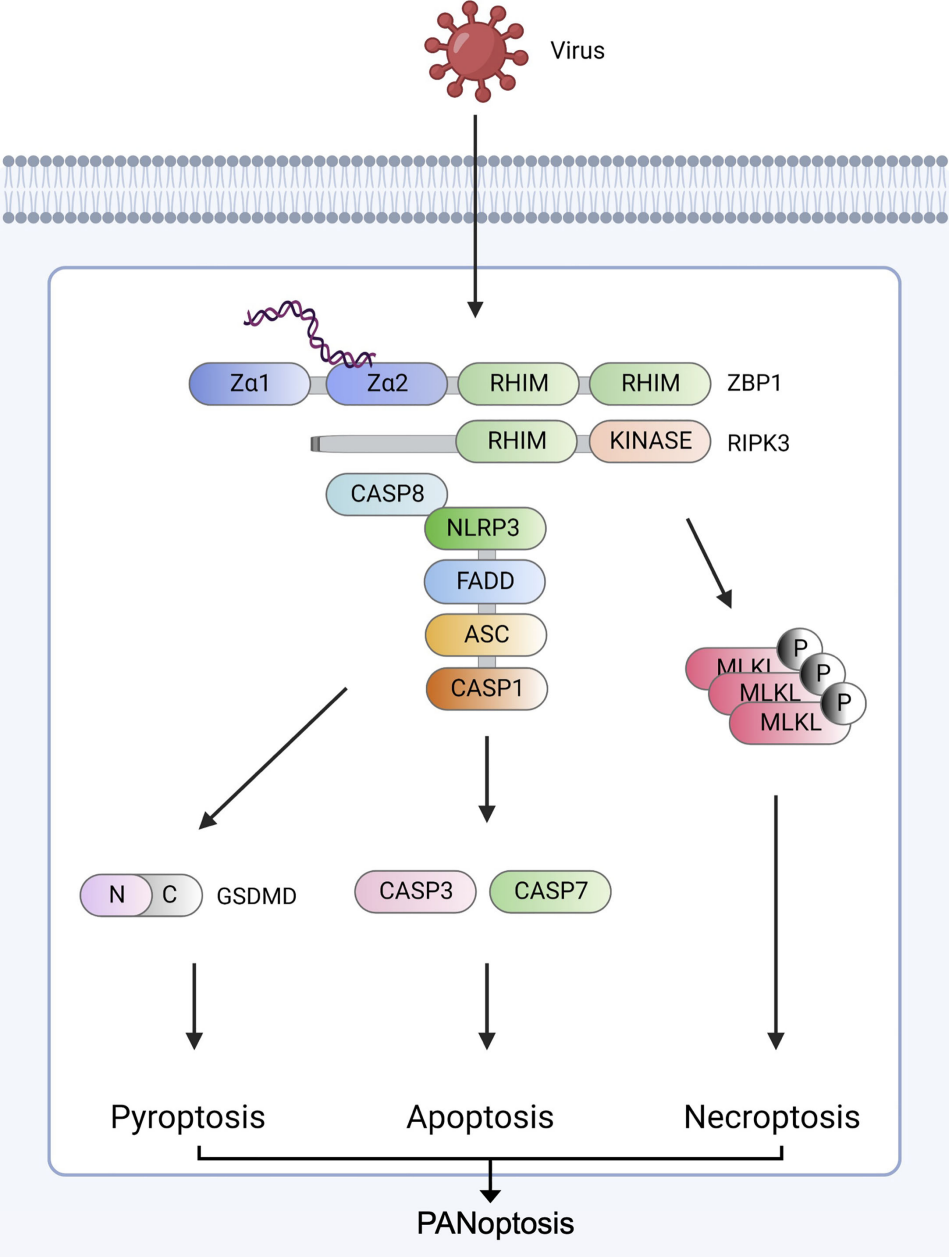
ZBP1-derived PANoptosis ZBP1 senses viral nucleic acids (Z-NA) and interacts with RIPK1 (not shown) and RIPK3 *via* RHIM domains to recruit caspase-8. The ZBP1-RIPK3-caspase-8 complex induces three major inflammatory cell death pathways: GSDMD-mediated pyroptosis, caspase-3/caspase-7-mediated apoptosis, and MLKL-mediated necroptosis. RIPK3 and caspase-8 induce NLRP3 inflammasome assembly *via* ASC and pro-caspase-1. After assembly, mature caspase-1 cleaves GSDMD to form pores in the plasma, leading to pyroptosis. Caspase-8 stimulates the secretion of caspase-3 and caspase-7. ZBP1-RIPK3 activates MLKL-mediated necroptosis. Created with BioRender.com.

The sensing of viral elements by the innate immune system induces inflammatory cell death pathways. After ZBP1 senses viral Z-RNA, a cascade of pro-inflammatory cytokines occurs, and PCDs are induced individually or together through crosstalk for host defense (**Figure 1**).

### ZBP1 induces pyroptosis during viral infection

Pyroptosis is a form of inflammatory cell death, typically mediated by inflammasomes. The term pyroptosis was first defined in 2001 semantically distinguishing pyroptosis and apoptosis (81). Canonical pyroptosis is regulated by inflammasome activation, which cleaves GSDMD and releases IL-1β and IL-18 (40, 41, 82). Gasdermin E (GSDME), another member of the gasdermin (GSMD) family, is also involved in pyroptosis *via* caspase-3 and -8 activation by undergoing cleavage and releasing N-terminus, thereby forming channels on the cell membrane (83, 84). The inflammasome is a multi-protein complex containing parts of a sensor, adaptor, and effector that assemble in response to the virus entry. The assembly of the inflammasome begins to sense certain stimuli through its sensor protein (85). For example, poly (dA:dT) is recognized by AIM2 (86), and NLRC4 detects Salmonella flagellin (87). Subsequently, pro-caspase-1 proteins form oligomers and activate caspase-1. Furthermore, the activated caspase-1 can proteolytically cleave the cytokines pro–IL-1β and pro–IL-18 into their bioactive forms to induce pro-inflammatory responses.

The relationship between the NLRP3 inflammasome and ZBP1 is well established. The ZBP1-NLRP3 inflammasome facilitates the maturation of pro-inflammatory cytokines, including IL-1β and IL-18, and GSDMD by activating caspase-1. IL-1β and IL-18 are processed into their active forms to upregulate the pro-inflammatory signaling pathway (88, 89). Simultaneously, GSDMD is cleaved by caspase-1 and it self-oligomerizes to form a pore in the membrane, releasing cytokines to induce inflammatory cell death through a process called pyroptosis (40–42). During IAV infection, ZBP1 activates the NLRP3 inflammasome and induces pyroptosis in BMDMs. Pyroptosis-associated cytokines, IL-1β and IL-18, were significantly reduced in *Zbp1^–/–^* BMDMs (11). In BMDMs, ZBP1-induced pyroptosis is regulated by RIG-I-MAVS and TLR signaling pathways during IAV infection (13). However, in MHV infection, *Zbp1^–/–^* mice survived more than WT mice after IFN-γ treatment (32). Similar to the ZBP1-NLRP3 inflammasome, the ZBP1-AIM2 inflammasome facilitates the expression of pyroptotic markers, such as caspase-1, GSDMD, and GSDME, and inflammasome activation is reduced in *Zbp1^–/–^* BMDMs in response to HSV-1 infection (18).

### ZBP1 induces apoptosis during viral infection

Apoptosis was first structurally distinguished from cell death and found to be involved in PCD in the development of *Caenorhabditis elegans* (90). Apoptosis is triggered by numerous stimuli, including viruses, and mediated by successive caspase reactions. This activation occurs *via* the *initiator* caspase, which is present upstream of the *effector* (or executioner) caspase. The apoptotic *initiator* caspase contains caspases-2, -8, -9, and -10, and *effector* caspases contain caspase-3, -6, and -7. These *effector* caspases play a central role in apoptosis by catalyzing their substrates.

ZBP1-associated apoptosis is also mediated by caspase-8, caspase-3, and caspase-7. Caspase-8 activates caspase-3, which promotes the maturation of GSDME to form pores in the membrane. Additionally, caspase-7 is activated by caspase-8. In *Zbp1^–/–^* BMDMs, caspase-8, -3, and -7 are downregulated during HSV-1 and IAV infection (11, 18). Additionally, the activation of caspase-8, -3, and -7 was attenuated in *Zbp1^–/–^* and *Zbp1* ^ΔZα2/ΔZα2^ BMDMs than in WT during MHV infection with IFN-β treatment (32).

### ZBP1 induces necroptosis during viral infection

Necroptosis and apoptosis differ in their morphology and molecular pathways. Apoptosis is characterized by cell shrinking, nuclear fragmentation, intra-nucleosomal cleavage, and membrane blebbing (91, 92). In addition, cells exposed to apoptosis show engulfment signals that are then detected by phagocytes. Necroptosis is characterized by a bursting membrane, cell lysis, and pro-DAMP release (93–95). Necroptosis is mediated by RIPK3, which interacts with other RHIM domain-containing molecules *via* the RHIM domain at the C-terminus (96). Similarly, ZBP1 induces necroptosis *via* the activation of RIPK3, which phosphorylates MLKL *via* its kinase domain. Phosphorylated MLKL is then inserted into the membrane, which constitutes a necroptotic pore. During HSV-1 infection, necroptotic markers, phosphorylated RIPK3 and MLKL, are reduced in *Zbp1^–/–^* BMDMs (18). The HSV1 viral protein ICP6 induces necroptosis in RHIM-RIPK3-MLKL dependent manner in ZBP1 deficient cells (97). After sensing IAV Z-RNA, ZBP1 induces RIPK3-MLKL-dependent necroptosis (98). In murine cytomegalovirus (MCMV) infection, ZBP1 regulates necroptosis with RIPK3, independent of RIPK1 (99). The phosphorylation of MLKL and RIPK3 was downregulated in the absence of the ZBP1 and Zα2 domains of BMDMs during MHV infection with IFN-β treatment (32). The Zα2 domain of ZBP1 senses vaccinia virus (VV) and induces necroptosis (100). In a study, after SARS-CoV-2 infection, the mRNA levels of ZBP1 and MLKL were increased in mouse neurons and brains (101). The expression of ZBP1, RIPK3, and caspase-8 was found to be increased in blood samples of patients with severe COVID-19, as analyzed using expression quantitative trait loci (eQTL) (102).

## ZBP1-PANoptosis: a double-edged sword

### PANoptosis

**Figure 2:**
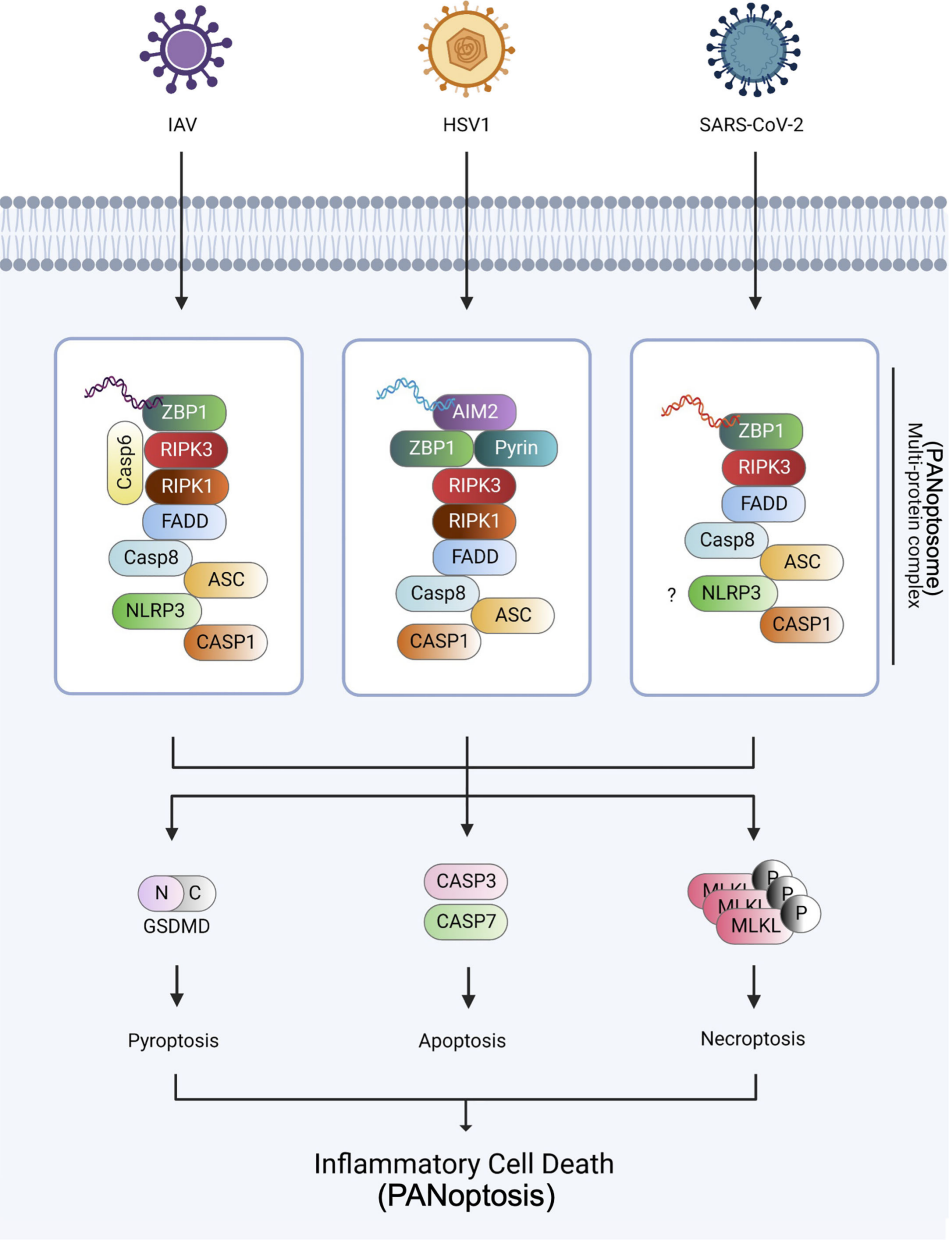
PANoptosome and PANoptosis against IAV, HSV1, and SARS-CoV-2 infections The three viruses, IAV, HSV1, and SARS-CoV-2, were established as the models of PANoptosis. During IAV infection, ZBP1 senses viral dsRNA and recruits RIPK3, RIPK1, caspase-6, and caspase-8 to assemble the PANoptosome, causing GSDMD-mediated pyroptosis, caspase-3 and -7-mediated apoptosis, and MLKL-mediated necroptosis. During HSV1 infection, AIM2, the dsDNA sensor, senses HSV1 dsDNA and recruits ZBP1 and pyrin during PANoptosis. During SARS-CoV-2 infection, ZBP1 interacts with the NLRP3 inflammasome *via* an unknown mechanism and induces PANoptosis. Created with BioRender.com.

PANoptosis is a unique inflammatory cell death process controlled by the PANoptosome, which reacts to specific stimuli, including viruses. The term PANoptosis was established based on studies that revealed a crosstalk between pyroptosis, apoptosis, and necroptosis. The crosstalk was first observed between pyroptosis and apoptosis (103, 104). Subsequently, the overlapping functions of caspase-8 and caspase-1/NLRP3 for pyroptotic, apoptotic, and necroptotic molecules were identified (61, 105, 106). ZBP1 (11), TAK1 (19, 107), and caspase-6 (14) were recently found to solidify the concept of PANoptosis. Additionally, the Zα2 domain of ZBP1 has been shown to be an essential component of IAV (30) and HSV1 (18)-induced PANoptosis. Furthermore, the roles of PANoptosis and cytokine storms in coronavirus infection have been studied (32, 37, 108). Overall, PANoptosis has been implicated in defense against various pathogens, including viruses (**Figure 2**).

### ZBP1 regulates PANoptosis as a central upstream molecule

ZBP1 has been investigated as a necroptotic sensor; however, recently, it has also been revealed to regulate multiple inflammatory cell death processes, including PANoptosis. The first study of ZBP1-derived PANoptosis reported that ZBP1 regulates NLRP3 inflammasome activation to induce PANoptosis *via* RIPK1-RIPK3-caspase-8 axis during IAV infection. This study suggests that ZBP1 is a central molecule that senses IAV infection by detecting the IAV viral proteins, NP and PB1 (11). IAV Z-RNA also induces PCD by activating ZBP1 and resulting in RIPK1 recruitment and caspase-8 activation (34). Furthermore, the Zα2 domain of ZBP1 regulates PANoptosis and NLRP3 inflammasome during IAV infection (30).

### ZBP1-PANoptosis defends host against viral infection

Several studies have established the *crosstalk* theory that explains the co-activation of pyroptosis, apoptosis, and necroptosis (PANoptosis). Since the first crosstalk between pyroptosis and apoptosis was revealed, ZBP1 showed a critical role in the crosstalk between inflammatory cell deaths. ZBP1 has been studied as a necroptotic sensor (99, 109). However, the regulatory role of ZBP1 was identified in multiple inflammatory cell death pathways. First, ZBP1 stimulates not only NLRP3 inflammasome activation but also apoptosis and necroptosis during IAV infection (11). This study suggests that ZBP1 is a key regulator of the three delegable inflammatory cell death pathways. ZBP1 was found to be highly expressed in IAV-infected WT BMDMs, while being downregulated in IAV-infected *Ifnar1^–/–^* BMDMs. IL-1β and IL-18 levels were reduced in *Zbp1^–/–^* BMDMs. The scientists observed an interaction between ZBP1 and RIPK3 using immunoprecipitation in IAV-infected WT BMDMs, which induced apoptosis and necroptosis during IAV infection (110, 111). Subsequently, the Zα2 domain of ZBP1 regulates influenza-inducible PANoptosis and NLRP3 inflammasome (30). The absence of the Zα2 domain of ZBP1 induced the downregulation of caspase-1 activation and GSDMD cleavage during IAV infection, which is a criterion for pyroptosis. The activation of caspase-8, caspase-3, and RIPK3 lacked *Zbp1* ^ΔZα2/ΔZα2^ BMDMs. These results indicated that the Zα2 domain of ZBP1 is critical for the activation of PANoptosis against IAV infection. Additionally, the absence of ZBP1 increases cell death. In summary, ZBP1 is beneficial for host cell survival against IAV infection (11, 32, 34). In other viral infections, such as HSV1, ZBP1 forms a complex with AIM2 and pyrin and defends the host *via* the induction of PANoptosis (18). In this study, ZBP1 cooperated with pyrin in AIM2 inflammasome activation during HSV1 infection, and ZBP1 induced PANoptosis in response to HSV1 infection in an AIM2-dependent manner. Additionally, a deficiency of the Zα2 domain of ZBP1 reduced HSV1-induced cell death and *Zbp1^–/–^* BMDMs. Overall, these studies suggest that ZBP1 is a central mediator of PANoptosis against viral infections to protect host cells from viral lethality.

### ZBP1-PANoptosis increases viral lethality through cytokine storm

**Figure 3:**
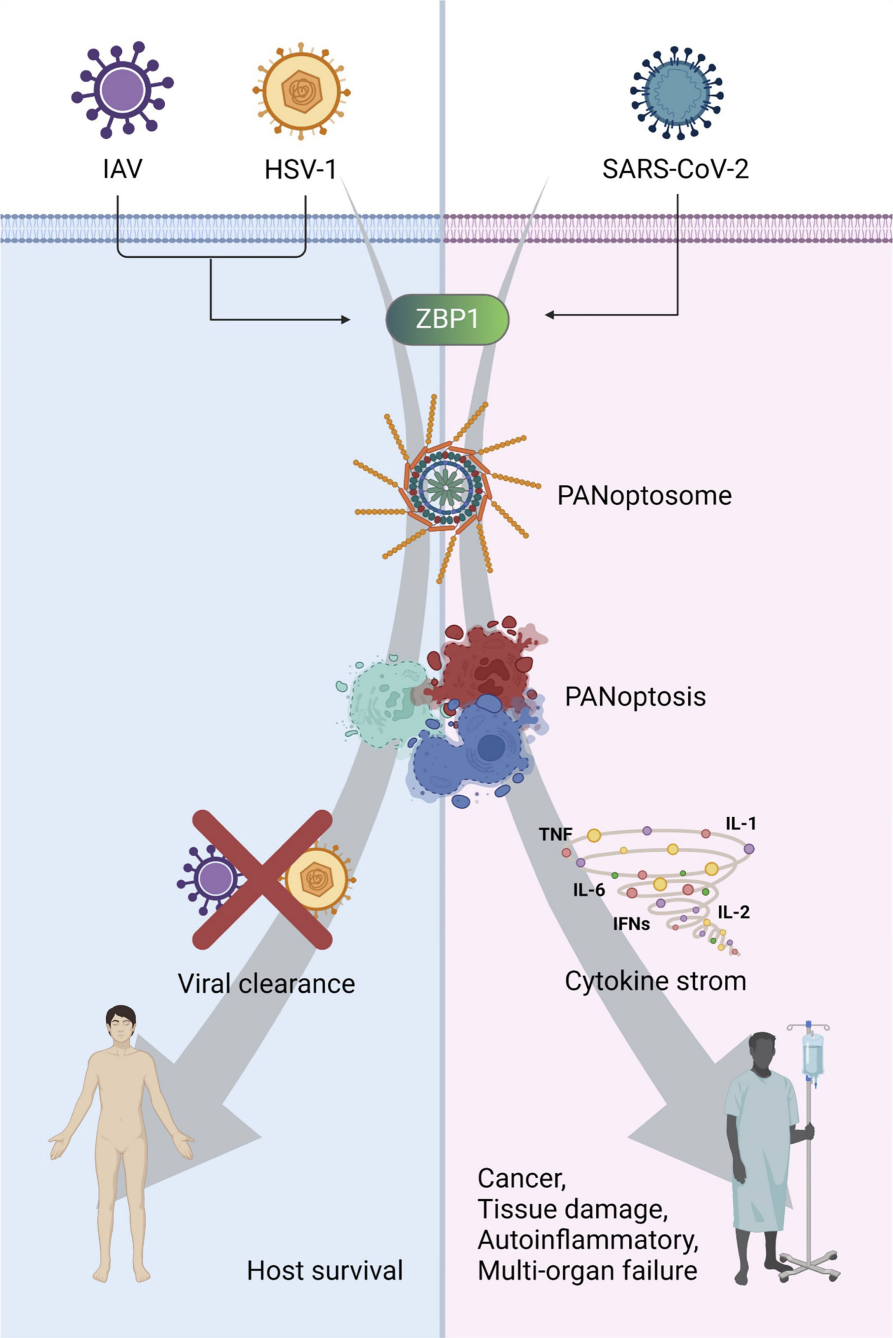
The consequence of PANoptosis: a double-edged sword in viral pathogenesis ZBP1-mediated PANoptosis can protect the host against viral infections, such as IAV and HSV1. However, it can trigger cytokine storms and cause cancer, tissue damage, autoinflammation, and multiple-organ failure. Created with BioRender.com.

PANoptosis is not always beneficial for host survival. Dysregulation of cytokines can cause cell death, tissue damage, and mortality due to viral infections (32, 38, 112). Immune hyperactivation occurs as an acute induction of pro-inflammatory cytokine secretion, resulting in a cytokine storm in β-coronavirus infections, including those of SARS-CoV (113–118), MERS-CoV (119–125), MHV (32, 108), and SARS-CoV-2 (32, 39, 126, 127). During SARS-CoV-2 infection, co-treatment with TNF-α and IFN-γ, which mimics the cytokine storm, induces PANoptosis *in vitro* and *in vivo*, viral lethality, and severe symptoms, such as tissue damage (37). Robust release of cytokines has been suggested to correlate with lung injury and multiple organ failure (**Figure 3**) (128–130). After screening a publicly available dataset, various pro-inflammatory cytokines were found to be upregulated in patients with severe COVID-19. Co-treatment with IFN-γ and TNF-α significantly induced PANoptosis in BMDMs and THP-1 cells *via* the STAT1-interferon regulatory factor 1 (IRF1)-inducible nitric oxide synthase (iNOS)-nitric oxide (NO) axis. NO induces apoptosis by activating caspase-8 (131, 132). Similarly, *Ripk3^–/–^Casp8^–/–^* BMDMs were rescued from PANoptosis induced by IFN-γ and TNF-α when compared with *Ripk3^–/–^* BMDMs. Moreover, *Ripk3^–/–^Fadd^–/–^* BMDMs were saved from PANoptosis induced by co-treatment with IFN-γ and TNF-α. Overall, the RIPK1-FADD-CASP8 axis induces PANoptosis by IFN-γ and TNF-α co-treatment. In the *in vivo* experiment, the levels of serum lactate dehydrogenase (LDH) and immune cells in the blood were reduced in *STAT1^–/–^* and *RIPK3^–/–^Casp8^–/–^* mice co-treated with IFN-γ and TNF-α. Blocking IFN-γ and TNF-α using neutralizing antibodies significantly increased the survival of SARS-CoV-2-infected mice when compared to that of an isotype control. Collectively, IFN-γ and TNF-α play critical roles in the induction of PANoptosis and cytokine storms during SARS-CoV-2 infection. Similarly, the Zα2 domain of ZBP1 upregulates PANoptosis and cytokine storm during β-coronavirus, SARS-CoV-2, and MHV infections with IFN treatment (32). Interestingly, delayed IFN-β release and STAT1 activation were observed in MHV infection, which mirrors the biology of human β-coronavirus. IFN-β treatment a few days after MHV and SARS-CoV-2 infection induces the activation of PANoptosis markers, including caspase-1, GSDMD, GSDME, caspase-8, -3, -7, MLKL, and RIPK3. Delayed IFN-β treatment influences the pathogenesis of MERS-CoV in a mouse model (3) and delayed IFN-α2b treatment upregulates mortality in patients with SARS-CoV-2 (133). These results indicate that delayed cytokine release favors β-coronavirus lethality. ZBP1 was then used to identify the sensing mechanism of β-coronavirus among ISGs significantly upregulated by MHV infection in immortalized BMDMs (iBMDMs). ZBP1-deficient mice showed no significant difference in the presence or absence of IFN-β, and ZBP1 was highly expressed in the lungs of mice infected with MHV following IFN-β treatment than in the lungs of MHV-infected and untreated mice. Similarly, a deficiency of the Za2 domain of ZBP1 reduces cell death during MHV infection. However, IFN-β treatment did not induce any changes in cell death during IAV infection in *Zbp1^–/–^* and *Zbp1* ^ΔZα2^ mice. Moreover, *Zbp1^–/–^* and *Zbp1* ^ΔZα2^ mice showed the downregulation of PANoptosis markers compared to WT mice during MHV infection with IFN-β treatment. In a very recent study, it was observed that the deletion of ZBP1 or RIPK3 reduced the secretion of inflammatory cytokines and chemokines and attenuated immune cell infiltration and lung damage during SARS-CoV-2 infection *in vivo* (39).

### Viral immune evasion

The virus can evade the host immune system through viral components including viral proteins. Viral immune evasion helps viruses grow, transmit, and survive in the host body to escape the host immune system, thereby causing failure in the immune response (134). The virus has diverse strategies for escaping the host immune system. They inhibit signaling pathways by targeting specific immune signaling-mediated proteins, such as inhibiting IRF9 (also known as p48) (135, 136) or blocking the phosphorylation of STAT1 (135, 137, 138). ZBP1 has also been reported as a target of viral evasion strategies (26, 99, 139, 140). An MCMV viral protein, M45, is considered to suppress the interaction of the ZBP1-RIPK1/RIPK3 and downstream signaling pathway depending on the RHIM domain (26), and ZBP1-RIPK3 interaction occurs in M45*mut*RHIM MCMV infection but not in WT MCMV (11, 99). This study identified ZBP1 as a target of MCMV evasion. VV viral protein, E3, which contains the Zα domain inhibits IFN and RIPK3-dependent necroptosis with ZBP1 during VV infection (140). Additional studies about viral evasion strategies are suggested from an RHIM-dependent perspective (141).

Other viral proteins may disrupt the inflammatory cell death signaling pathway. The nonstructural protein (NS1) is a well-known IAV viral protein that inhibits the transcription of antiviral genes and intracellular ISGs, including protein kinase R (PKR) (142) and 2,’ 5’-oligoadenylate synthetase (2’-5’ OAS), by binding to viral RNA to prevent detection by ISGs (143). The other IAV viral protein, PB1, particularly PB1-F2, directly interacts with MAVS (144, 145). PB1-F2 induces pyroptosis by interacting with the NLRP3 inflammasome, which causes an increased production of IL-1β (146). Additionally, the substitution of an amino acid (Asn66Ser) is known to inhibit type 1 and type 3 IFNs (147). Similarly, HSV can evade the host immune system through various mechanisms. Similar to IAV, HSV represses the IFN response through viral proteins, including ICP0, ICP27, ICP34.5, Us3, and vhs. Each protein inhibits IFN expression in diverse ways. ICP0 modifies IRF3 and IRF7 (148, 149), and ICP27 reduces IFN and cytokine expression by inhibiting IRF3 and NF-kB activation (150). Downstream molecules of the IFN signaling pathway, such as STAT1, are also targeted by HSV viral proteins. In addition, HSV viral proteins ICP4, ICP27, ICP34.5, and gJ inhibit apoptosis in various ways, such as caspase inhibition and downregulation of Fas ligand (151). Coronavirus impedes the innate immune system using viral proteins, nonstructural proteins (Nsp), and open reading frames (OFR). Nsp1 inhibits the IFN signaling pathway, particularly SARS-CoV-2 Nsp1, which suppresses the promoter activity of IFN-stimulated response elements (ISREs) (152). Nsp3, the largest protein encoded by the coronavirus genome, can bind to IRF3 and suppress the phosphorylation and nuclear translocation of IRF3, thereby leading to the inhibition of the IFN signaling pathway (153). Moreover, Nsp13 and Nsp15 can modify the viral RNA to escape from the guards of the host. The 5’-ppp moiety, a type of RIG-I ligand, is regulated by Nsp13 (154, 155), and Nsp15 removes the 5’-polyuridine (polyU) region from 5’-polyU-containing, negative-sense RNAs, which helps viral RNA to hide from cytosolic dsRNA sensors, including PKR, MDA5, and OAS/RNase L (156). The ORF family also antagonizes the host inflammatory and IFN signaling pathways (157–167). These numerous strategies allow viruses to evade the host innate immune system, particularly the IFN signaling pathway, and these strategies may evolve owing to the importance of IFN in defending the host from viruses.

### Cytokine storm-related cytokines: IFNs and TNF

IFNs and TNF are essential components of the innate immune system. IFN secretion is activated when PRRs (RIG-I) sense viral components and stimulate IRF3, which induces the secretion of type 1 IFNs. Secreted IFNs are then detected by IFNAR1/2, and the STAT1 signaling pathway is induced (80, 168). ZBP1 is also a downstream molecule in the IFN pathway. Induction of IRF9 by the STAT1 pathway stimulates ZBP1 expression in *Ifnar1* ^–/–^, *Stat1* ^–/–^, and *Irf9* ^–/–^ cells, and ZBP1 activation was abolished (11). IFNs are required to induce ZBP1-derived inflammatory cell death. IFNs mediate pyroptosis (11, 169), necroptosis (170), and apoptosis (171) associated with ZBP1. Therefore, IFN is critical for PANoptosis. Cytokine storms, which compensate for severe viral lethality in the host, are also related to IFNs. One of the well-studied theories about the relationship between IFNs and cytokine storms is the delayed activation of IFN. IFN delay enhances cytokine secretion and disease during viral infections (3, 133, 172). During MERS-CoV infection, IFN-β delay enhances pro-inflammatory cytokines released in monocytes, macrophages, and neutrophils (3). Delayed type 1 IFN signaling promotes SARS-CoV infection (172). Besides, CD4^+^ and CD8^+^ T cells, which are involved in adaptive immune, are reduced in severe COVID-19 (173, 174), and Th17 CD4^+^ T cells, which act in a pro-inflammatory role, are increased (126). In a recent study, SARS-CoV-2 revealed that can directly infect T lymphocytes in a spike-ACE2/TMPRSS2-independent manner (175). The dysregulated immune system induces non-specific immune cells and the release of pro-inflammatory factors (176, 177). In conclusion, the IFN secretory pathway may burst during the late phase of viral infection and promote host survival.

## Concluding remarks

In this review, we summarize the molecular-based mechanism of inflammatory cell death by focusing on viruses that cause all three major cell deaths. Considering IAV and HSV1 infections, ZBP1-derived PANoptosis plays an important role in host survival. However, side effects such as a cytokine storm in coronavirus infections lead to systemic inflammation, organ failure, and even death of the host. Therefore, virus-induced cell death must be controlled to reduce the hyperactivation of the immune response by conducting virus-specific studies. There are differences in the viral infection method, viral life cycle, and immune evasion strategies for each virus. In addition, various viral proteins help viruses evade and cause confusion in the host immune system. Further studies should be conducted to elucidate the exact mechanism through which the cytokine storm occurs.

The regulation of cytokines and signaling pathways is important for controlling the pathogenesis of the hyperactivated immune system. In particular, TNF and IFN-γ synergize cytokine storms by generating a feedback loop in coronavirus infection. Thus, the neutralization of TNF and IFN-γ may be valuable to rescue excessive cytokine secretion (178). This process is induced by the caspase-8-JAK1/2-STAT1 axis (178). Inhibitors of molecules that participate in this signaling pathway, such as STAT1 or JAK1/2 inhibitors, would be effective. In the case of JAK1/2 inhibitors, baricitinib received an emergency use authorization to cure COVID-19 in 2020. Collectively, pro-inflammatory cytokines must be regulated to rescue the host from the cytokine storm loop and subsequent lethal symptoms, such as systemic inflammation.

Other strategies that target inflammasome components, including sensors, ZBP1, NLRP3, and downstream molecules, caspases, RIPK1, RIPK3, and ASC may be helpful. In caspase-8 and MLKL double-knockout (DKO) mice, weight loss induced by SARS-CoV-2 infection was abolished, although the viral burden did not change (179). *Casp8* ^–/–^ *Ripk3* ^–/–^ DKO mice were rescued from viral lethality induced by TNF and IFN-γ co-treatment but not *Ripk3* ^–/–^ mice (37). These results indicate that the components of PANoptosis play an important role in fatal cytokine storms. If we can directly control ZBP1 and NLRP3, this may be an efficient method. One of the possible strategies is by using ADAR1. ADAR1 acts as a repressor of the ZBP1-NLRP3 inflammasome and causes multiple inflammatory cell deaths (33). Additionally, there are other studies on the capacity of ADAR1 for suppressing ZBP1-mediated PCD (180–183). Collectively, we can overcome ZBP1-derived multiple inflammatory cell death and severe signs, such as cytokine storms, using molecular-based therapeutic strategies.

Furthermore, IFN delay causes hyperactivated secretion of cytokine (3, 133, 172). Thus, IFN therapy, which is used for the treatment of viral infection, would be harmful to patients with COVID-19 by eliciting excessive activation of cytokines. Therefore, treatment should be administered with caution after further research. In summary, the unresolved questions need to be addressed to develop a strong defense strategy against viral infections and to control multiple inflammatory cell deaths.

## Author contributions

SO and SL conceived the manuscript. SO and SL wrote the manuscript. SO and SL critically revised and approved the final version of the manuscript. All authors have contributed to the manuscript and approved the submitted version.
